# Computer-Aided Diagnosis of Coal Workers’ Pneumoconiosis in Chest X-ray Radiographs Using Machine Learning: A Systematic Literature Review

**DOI:** 10.3390/ijerph19116439

**Published:** 2022-05-25

**Authors:** Liton Devnath, Peter Summons, Suhuai Luo, Dadong Wang, Kamran Shaukat, Ibrahim A. Hameed, Hanan Aljuaid

**Affiliations:** 1 School of Information and Physical Sciences, The University of Newcastle, Newcastle, NSW 2308, Australia; peter.summons@newcastle.edu.au (P.S.); suhuai.luo@newcastle.edu.au (S.L.); 2 Quantitative Imaging, CSIRO Data61, Marsfield, Sydney, NSW 2122, Australia; dadong.wang@data61.csiro.au; 3 Department of Data Science, University of the Punjab, Lahore 54890, Pakistan; 4 Department of ICT and Natural Sciences, Norwegian University of Science and Technology, 7491 Trondheim, Norway; 5 Computer Sciences Department, College of Computer and Information Sciences, Princess Nourah bint Abdulrahman University (PNU), P.O. Box 84428, Riyadh 11671, Saudi Arabia; haaljuaid@pnu.edu.sa

**Keywords:** coal workers’ pneumoconiosis, computer-aided diagnostic, occupational lung disease, pneumoconiosis, black lung, texture feature analysis, machine learning, deep learning, chest X-ray radiographs, systematic literature review

## Abstract

Computer-aided diagnostic (CAD) systems can assist radiologists in detecting coal workers’ pneumoconiosis (CWP) in their chest X-rays. Early diagnosis of the CWP can significantly improve workers’ survival rate. The development of the CAD systems will reduce risk in the workplace and improve the quality of chest screening for CWP diseases. This systematic literature review (SLR) amis to categorise and summarise the feature extraction and detection approaches of computer-based analysis in CWP using chest X-ray radiographs (CXR). We conducted the SLR method through 11 databases that focus on science, engineering, medicine, health, and clinical studies. The proposed SLR identified and compared 40 articles from the last 5 decades, covering three main categories of computer-based CWP detection: classical handcrafted features-based image analysis, traditional machine learning, and deep learning-based methods. Limitations of this review and future improvement of the review are also discussed.

## 1. Introduction

Pneumoconiosis is an occupational lung disease and a group of interstitial lung diseases (ILD) caused by chronic inhalation of dust particles, often in mines and from agriculture, that can damage both lungs and is not reversible [1,2,3]. There are three important occupational lung diseases, coal worker pneumoconiosis (CWP), asbestosis, and silicosis, seen in Australia [4]. CWP (commonly known as black lung (BL)) is mainly caused by long-term experience with coal dust, which is similar to silicosis lung disease caused by silica and asbestos dust. Pneumoconiosis, including CWP, asbestosis, and silicosis, killed 125,000 people worldwide between 1990 and 2010, according to the Global Burden of Disease (GBD) [5]. The national mortality analysis from 1979–2002 reports that over 1000 people have died in Australia due to pneumoconiosis, with CWP, asbestosis, and silicosis representing 6%, 56%, and 38% of the total, respectively. Pneumoconiosis has increased due to poor dust control and a lack of workplace safety measures [6,7,8,9].

In clinical imaging, computer-aided diagnosis (CADx), also known as computer-aided detection (CADe), is a system developed for the computer to help make quick decisions for future treatment [10,11]. Medical image analysis is now an essential assessment for detecting possible clinical abnormalities at an earlier stage. CAD systems help to improve diagnostic image systems, visualising suspicious parts and highlighting the most affected area of images in X-rays, CT-scans, ultrasounds, and MRI [12,13].

CAD systems have been in use for more than 45 years and have multiple applications in artificial intelligence and computer vision problems. Even though the CAD process is well established in radiology, it is not an alternative to a clinician but is used as an aid that helps radiologists to make better decisions. There is no automated way to detect and screen for the CWP, except for radiologists who specialize in the field. The lack of an available CWP database presents significant challenges to developing an automatic screening system. This is due to the low prevalence of CWP and restrictions on the sharing of patient data.

There are several reviews on pneumoconiosis among coal miners; most of them discussed the prevalence, risk factors, survival rate, and prevention of CWP based on the pathological findings [7,14,15,16,17,18,19,20,21,22,23,24,25,26]. The past CAD studies of CWP disease indicate no significant review of machine learning applications that might help improve future work in the area. In this paper, we present a unique systematic review of contemporary approaches to CAD for CWP in chest radiographs through classical, traditional, and deep learning approaches, which will indicate the successes, limitations, and possible future directions of research.

Section 1.1 overviews the international labor organisation’s (ILO) standard guideline of pneumoconiosis classification. Section 2 provides the proposed method for this systematic literature review (SLR), the search strategy, database, and study selection. Section 3 reveals the study results in an integrated framework, including the study context and classification in terms of three feature analysis methods, texture analysis, opacity measurements, non-textured analysis, and their detection approaches through classical, traditional, and deep learning. Section 4 compared the CAD performance between the identified patterns of feature analysis and detection approaches. Finally, we found some limitations and suggested future directions for CWP detection in Section 5.

### 1.1. Standard Classification of Pneumoconiosis

The abnormality on a chest X-ray of the lung is signified by an increase or decrease in density areas. The chest X-ray lung abnormalities with increased density are also known as pulmonary opacities. Pulmonary opacities have three major patterns: consolidation, interstitial, and atelectasis. Among them, interstitial patterns of pulmonary opacities are mainly responsible for BL disease [27,28,29]. According to ILO classification [30], there are two types of abnormalities, parenchymal and pleural, seen for all types of pneumoconiosis, such as our target research topic BL disease or CWP.

The ILO has categorised pneumoconiosis into 0, 1, 2, and 3 stages, where 0 is normal and 3 is the most complicated stage of the disease. The stage of the disease is indicated by the profusion of small and large opacities, which may be round or irregularly shaped, which presents the parenchymal abnormality. The ILO classifies the size (diameter) of small rounded opacities as p, q, or r, indicative of diameters: p≤1.5, 1.5≤q≤3, 3≤r≤10 mm and defined by the presence on the six significant zones (upper, middle, lower) in both left and right lungs. On the other hand, the size (widths) of small irregular opacities is illustrated by the letters shown in standard radiograph areas, s≤1.5, 1.5≤t≤3, 3≤u≤10 mm. Opacities with a dimension of more than 10 mm are defined as large opacities. They are divided into three major categories, defined as 0≤A≤50 mm, 50≤B≤Area(RUZ), and 50≤C≥Area(RUZ), where RUZ indicates the area of the right upper zone (RUZ). 

**Figure 1:**
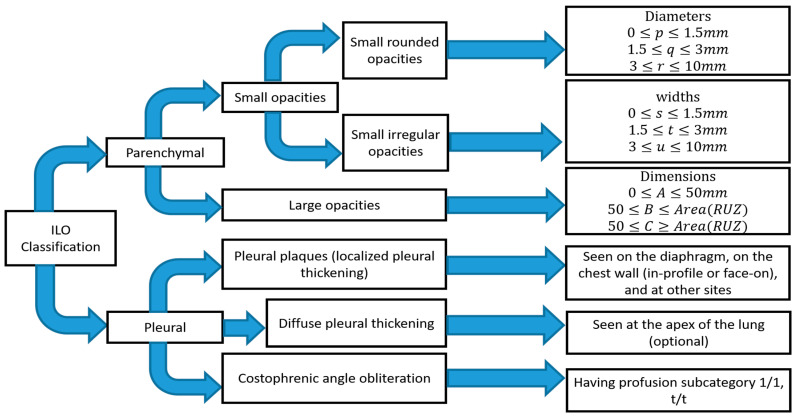
Summary of ILO standard classification of pneumoconiosis.

In pleural abnormalities, the ILO has shown that the parietal pleura is seen in the chest wall, diaphragm, and other sites of the lungs that can diffuse the thickness and decay at the appropriate angle of the lung frame. Figure 1 summarises parenchymal and pleural abnormalities, followed by standard opacities and their perfusion measurements. 

It is difficult for radiologists to classify pneumoconiosis in both types of abnormalities. The measurement of the size and shape of all regular and irregular opacities is quite difficult, especially in the earlier stage of CWP disease [31]. The radiographic changes in some blood vessels forming the opacities’ shape and the size of pneumoconiosis are difficult to diagnose. In addition, pleural plaque on plain chest radiographs shown in the shadows of ribs may lead to misclassification of conditions consistent with pneumoconiosis [32,33]. Therefore, the development of significant computer-aided diagnosis (CAD) schemes is necessary to reduce the risk in the workplace and improve the chest screening for pneumoconiosis diseases.

## 2. Method

This paper’s systematic literature review (SLR) method consists of three main sections, namely planning, conducting, and reporting. The planning section presents the necessity for the SLR, the review questions and protocol, and the evaluation of the protocol. In the conducting section, inclusion and exclusion criteria are defined and applied to select the appropriate literature for inclusion in the review. In the reporting section, the findings of the articles considered for inclusion in the review are reported, and their results are discussed, including details on feature analysis and detection approaches. 

This SLR compares the state-of-the-art for CAD diagnosis of CWP detection in chest X-ray radiographs based on three detection approaches, classical, traditional machine learning, and deep learning. To keep the SLR focused, an overarching research question was defined: 

“What is the current body of machine learning to detect CWP in CAD systems using chest X-ray radiographs?”

### 2.1. Search Strategy and Database Selection

Three components form the overarching research question were identified: domain (‘Black Lung’), trigger (‘Machine Learning’), and action (‘Chest X-ray’). Using these components in an initial search string, seven highly relevant papers from a web search (through GoogleScholar) were selected. Studying these papers and proceeding through several iterations, several other relevant keywords were identified and added to the initial search string. Another component was added to the search string to account for the agent (e.g., human, user). After analyzing the similarities and relevance of the phrases in each of the search string components, the final search string was refined to:

(“Black Lung” OR “Coal Workers Pneumoconiosis” OR Pneumoconiosis) AND (Detection OR Diagnosis OR “Computer-Aided Diagnosis” OR Identification OR Classification OR Analysis) AND (“Chest” OR “X-ray” OR “Radiographs” OR “Chest-X-ray-Radiographs”) AND (“Machine Learning”, “Deep Learning” OR “Convolutional Neural Networks “ OR “Neural Network” OR “Artificial Neural Network” OR “Support Vector Machine” OR “k-nearest neighbor”)

To address the research question, 11 databases that focus on science, engineering, medicine, health, and clinical studies, respectively, were selected for the review: Science Direct, Scopus, Springer Link, Pubmed, Medline, IEEE, Embase, Web of Science, Compendex, ACM, and CINAHL.

### 2.2. Study Selection Criteria

**Figure 2:**
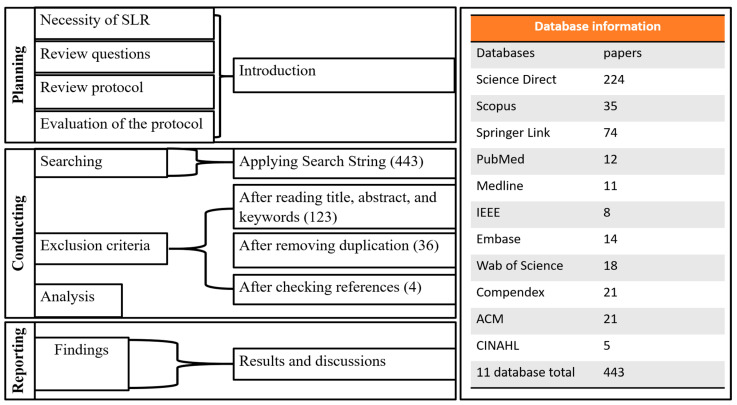
Overview of proposed SLR.

The SLR process and results of applying the search string and filtering for relevance are shown in Figure 2. Firstly, the search string was applied using the advanced search guideline for each of the eleven databases. Although all keywords remained the same, a few modifications were required, resulting in 443 articles across the eleven databases. Secondly, the title, abstract, and keywords of all search results were read to find relevance for this review, resulting in 123 articles. After removing duplications, the full text of the remaining articles was read, resulting in 36 papers that were the most relevant to the research question. When all references in the 36 papers were checked, four more relevant papers were added, increasing the total number of reviewed papers in this SLR to 40 (see Figure 2).

## 3. Study Results

The systematic literature review found four hundred and forty-three studies in the literature, forty of them meeting the criteria for inclusion. Returned articles were from 1974 to 2021, focusing mainly on pneumoconiosis in coal workers, and included three detection approaches, classical, traditional machine learning, and deep learning methods. The Analysis of Returned Articles section (Section 4) discusses these in detail. After reviewing the final selection of 40 papers, three interesting patterns were identified. The papers were divided into categories illustrating those patterns: CAD with texture analysis, opacity measurements, and non-texture analysis. These are discussed in the Analysis of Returned Articles (Section 4.1, Section 4.2 and Section 4.3, respectively).

**Figure 3:**
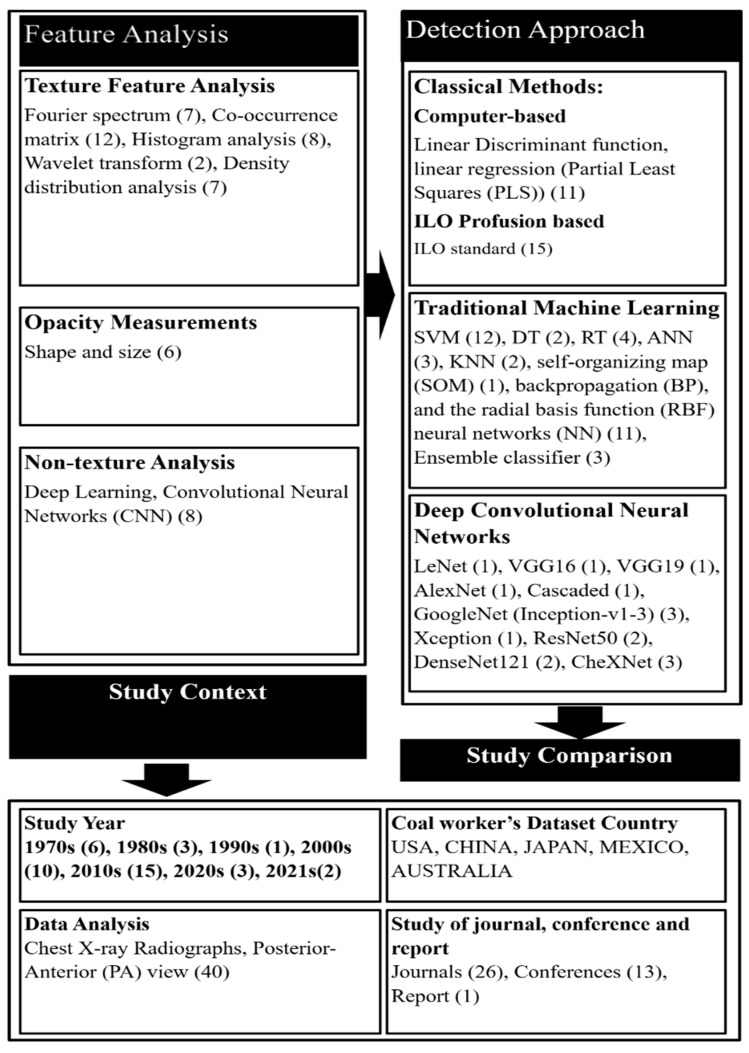
Theoretical framework for the CAD of CWP based on machine learning.

In texture analysis, different statistical approaches were applied to extract features from chest X-ray radiographs. The size and shape of round opacities were measured from regions of interest (ROI) identified in chest X-ray radiographs. In non-textured analysis, deep convolutional neural networks (CNN) were applied to chest X-ray images. Figure 3 presents articles categorised by these CAD framework approaches. It indicates the SLR article publication year and the coal mining dataset’s country. 

From the overall study of the 40 papers, 26 were classified in the texture analysis category, 6 papers in opacity measurements, and 8 papers in non-textured analysis categories. Five major categories of texture feature analysis methods were found; they are summarised in Table 1 and are discussed in detail in Section 4.1. The study’s theoretical framework (Figure 3) indicates the number of publications included based on their year of publication and type (journal, conference, or report). The context also indicates that only one type of chest X-ray radiograph view, called the posterior–anterior (PA), was used throughout the literature. The coal workers’ data were collected from different countries, including the USA, China, Japan, Mexico, and Australia.

Investigation of the methodology and detection approaches used in the reviewed articles also identified three detection approaches: classical methods including computer and international labor organization (ILO) classification-based detection; traditional machine learning methods; and CNN methods. A summary of studies categorised based on their detection approaches is presented for classical approaches (Table 2), traditional machine learning (Table 3), and deep learning (Table 4). In Table 2, Table 3 and Table 4, the country of origin of the datasets is indicated, with the USA shown as U, China as C, Japan as J, Mexico as M, and Australia as A. It is important to note that many of the papers do not share all of the values that were sought for this analysis. As a result, we could not incorporate these missing values into Table 2, Table 3 and Table 4. On the other hand, our aim was to draw attention to the specific number of CWP radiographs, feature analysis methods, and detection approaches used in their study. 

## 4. Analysis of Returned Articles

This section discusses the results and analysis of the articles returned in the study. The three patterns identified in the returned study articles from Section 3, CAD with texture analysis, opacity measurements, and non-texture analysis, are discussed in Section 4.1, Section 4.2, and Section 4.3, respectively. Section 4 details the three detection methods found in the returned articles from Section 3; classical methods (including computer and ILO-based detection), traditional machine learning, and deep learning (CNN-based) methods, discussed in Section 4.4.1, Section 4.4.2 and Section 4.4.3, respectively.

### 4.1. Datasets

Texture analysis was the key use of CAD of CWP in chest X-ray radiographs in the past year. A set of texture elements with regular or irregular patterns is called an image’s texture and represents the spatial arrangement of intensities in a particular region. The measurements of those arrangements are known as texture feature vectors of that region [74]. In medical imaging, texture analysis plays a vital role in finding object, defect, and pattern of an image. There are four types of texture analysis methods, statistical-based, model-based, transform-based, and structural-based, used in the literature [75]. The CAD of pneumoconiosis especially focuses on statistical and transform-based texture analysis methods [76,77,78]. 

The study found five major categories of texture feature analysis methods, where texture feature was extracted using Fourier spectrum [39,40,44,48,50,53,58], co-occurrence matrix analysis [42,48,50,53,55,57,58,59,61,64,79], histogram analysis [34,47,50,55,59,61,63,66], wavelet transform [52,56], and density distribution [42,45,46,51,54,60,62]. The details of the five methods are discussed in the following subsections and the texture features extracted from them, as described in Table 1, are summarised.

#### 4.1.1. Fourier Spectrum Analysis

In 2D images, an essential small unit presents the local information of a given pixel, and the texture spectrum indicates the frequency distribution within those units [74,80]. It also signals that a similar texture unit categorises every local texture of the given pixel. The popular Fourier transform (FT) of a particular region of interest (ROI) of an image transforms spatial information into a frequency domain where the spectrum contains the uniform texture image as well as its position [81]. The texture power spectrum (PS) is measured using different size ROIs [50,53,58] by Fourier transform, where different enhancement methods are used before calculating PS values. Recently, Katsuragawa et al., Turner et al., and Kruger et al. have also applied the FT with the visualisation of texture patterns for the diagnosis of CWP in CXR [40,44,48]. The RMS (root mean square) variation and momentums (first to third) values from the PS of abnormal lung ROIs are noticeably separated from normal lungs [39]. Ledley et al., used simulation software to produce texture characteristics that give geometrical size and shape formats in a grey-level image. The descriptions of Fourier spectrum-based features can be found in Table 1.

#### 4.1.2. Co-Occurrence Matrix Analysis

A co-occurrence matrix is an n-dimensional spatial arrangement. Each pair of rows and columns represents the possible pixel intensity (greyscale tone or colour tone) of an image [74]. The matrix is also referred to as the grey-level co-occurrence matrix, grey-level co-occurrence histogram, and spatial dependence matrix. In 1973, Haralick et al. [82] recommended 28 features extracted from the co-occurrence matrix for image classification. In the last few decades, these features have hardly been used in many CAD systems. This review also noticed that in the CAD of pneumoconiosis, texture features were extracted using grey-level co-occurrence matrix, grey-level co-occurrence histograms and spatial dependence matrix [42,48,50,53,55,58,59,61,64,65]. This study observed that texture features, correlation, contrast, homogeneity, entropy, and energy were mainly used to detect CWP in the chest X-ray radiographs (CXR). Their descriptions can be found in Table 1.

The texture feature In CXR is significantly affected by the quality of film images, which indicates the structural pattern difference between ribs and veins, which are not mentioned in the ILO standard of pneumoconiosis classification based on perfusion of opacities. Kobatake et al. [42] extracted the features of a film CXR dataset in matrix values, correlation, contrast or inertia, homogeneity, entropy, and energy which were also proposed by Kruger et al. [48]. In 2017, Okumura et al. [83] followed up their previous work in [84,85], which also extracted similar feature patterns from all ROIs by Fourier transform in different directions of the gradient vectors. In 2013, Cai et al. [55] tested the different texture features, including the co-occurrence matrix, as did Yu et al. [59,64]. This study also found that the CAD performances on grey-level co-occurrence features in [59,64] are more noticeable than those of Cai et al.

#### 4.1.3. Histogram Analysis

The histogram of an image represents the total tonal distribution of pixel values within the image. The tonal distribution indicates the variation of colours, especially grey-level intensity distribution, measured from histogram analysis [86,87]. In CAD of CWP diseases, several researchers have proposed extracting texture features using histogram analysis [34,47,50,55,59,61,63,66]. Most of them were computed using a set of common features, namely, mean, variance, skewness, kurtosis, energy, and entropy, from the grey-level intensity distribution of ROIs images. Their descriptions can be found in Table 1.

For the abnormality of CWP in CXR, where different sizes of angular opacities are present, better image enhancement techniques highlight small, round, regular, and irregular opacities [35,88,89]. The multi-scale difference filter bank was also used in [59,61,63] before histogram features were extracted, which improved the image contrast from different angles. Murray et al. [34] proposed a partial least squares approach for detecting CWP, where a multi-scale bandpass filter was used to extract features from histograms based on the different amplitudes and frequency-based representation which computed the different grades of opacities.

#### 4.1.4. Wavelet Analysis

In statistical analysis, multiresolution techniques refer to transforming an image into another presentation in which multi-scale statistics are presented. Because the texture format presents so many difficulties, wavelet analysis is a very popular method to visualise textural features in a multi-scale format. The discrete wavelet transformation with decomposition scales one decomposed image into four sub-bands representing the finest wavelet coefficients that are the essential features. Both single and combined values of sub-band images act as features [90,91,92]. The energy feature is an integrated value of single or combined decomposed images extracted in [52,56] for CWP detection in CXR. The descriptions of energy features can be found in Table 1—only two articles related to wavelet-based texture feature analysis in CAD of pneumoconiosis disease. Zhu et al., proposed the 2D tree structural wavelet decomposition [93] for the first time in their article from 2013 [56] and 2014 [52]. They used a maximum of twenty-eight wavelets and seven scale tree decomposition to extract energy texture features from sub-band full-size images. For better classification, they input the logarithmic values of energy features into different traditional machine learning classifiers.

#### 4.1.5. Density Distribution Analysis

In statistical analysis, the distribution of density among the textural features of a co-occurrence and histogram matrix is also a method of CAD systems. This study found literature related to the density matrix of distribution of the gradient vector on film CXR [42,45,46]. Here, the gradient vectors demonstrate the rates of density variation in different directions. The rapid increase in the density of a region indicates the profusion of opacities. An image’s texture varies depending on the scanner’s quality, which is very expensive in the clinical diagnosis system. To address this issue, Abe et al. [51,54] and Nakamura et al. [60,62] proposed a charge-coupled device (CCD) scanner for CAD of pneumoconiosis in CXR, where they computed the feature characteristics based on density distribution in a particular region or the areas between the ribs and its inter-costal. Their descriptions can be found in Table 1. They found the different feature characteristics of abnormal CXR, which execute the classifier performance.

### 4.2. Opacity Measurement

The CAD of coal mining disease also relies on analyses based on the evaluation and measurement of the size and number of small round opacities [36,37,38,41,43,94]. Small round opacities may appear anywhere in the lung and overlap within rivers and blood vessels. An image enhancement is applied to the CWP lung in which the grey-level difference indicates the round opacities [41,43]. The method highlights that the density of the lung opacity area is less than its surrounding background in an image.

In automatic detection of CWP from CXR, the redundant parts of the lung are a problem, especially in detecting of small round opacities. Kondo et al., proposed a moving normalisation technique to overcome this issue, which removed the redundant parts, such as ribs and blood vessels, in the ROI image. As a result, the small round opacities are visible in each ROI, and classification was done based on the size and number of opacities according to ILO standards [36,37,38,94].

### 4.3. Non-Texture Analysis

In the automatic diagnosis of CWP, several models have been developed in recent years based on deep convolutional neural networks (CNN), a family of deep machine learning methods. Every CNN has two parts: feature extraction and classification [95]. In the feature extraction part, several convolutional blocks are composed of a set of polling and activation functions. The deep CNN features of different classes are classified using one or more fully connected layers [95,96,97]. In the most recent computer vision applications, CNN has been used in many fields, including medical image analysis, which achieved outstanding state-of-the-art performances [98,99]. This study only found eight research articles based on the use of CNN to detect CWP in CXR in which non-texture features were extracted from the lung image [49,67,68,69,70,71,72,73]. Zheng et al. [73] investigated the CAD of CWP with the CXR films dataset, which indicated that traditional texture analysis is not enough to diagnose. They applied a pre-trained CNN model, GoogleNet [100], to classify normal and different stages of abnormal X-ray films where three scales of convolutional kernels improve the abstract feature quality. They also verified the non-textured feature performances with older versions of CNN, for example, LeNet [101] and AlexNet [102]. Zhang et al. [67] investigated the non-textured feature’s performances with two groups of radiologists. They found that the ResNet [103] model extracted the proper features from the six sub-regions in the lung, which outperformed the radiologists.

Arzhaeva et al. [49] developed a new CNN model, applying cascade learning to the automatic detection of pneumoconiosis, in which the model achieved the best accuracy compared to other statistical and traditional machine learning approaches. To address the dataset limitation, they used two augmented techniques which improved the deep CNN quality and increased the performance [104,105]. Dadong et al. [69] employed the same enhanced techniques as [105] to increase training data. Therefore, their 15-layer CNN model was used to extract features from the augmented train data and then evaluate the efficiency in non-augmented test data. Transfer learning improves CNN with small datasets in recent applications. Transfer learning is a method of transferring knowledge from one class to another that has similar characteristics. This saves time and aids in deep learning training on small datasets. Due to the unavailability of the dataset, Devnath et al. [72] at first investigated the efficiency of CNN with and without transfer learning for the detection of black lung disease in CXR, with the result that transfer learning with CNN was found to be a good approach [106,107,108]. Therefore, they proposed a comparison of seven CNN models, VGG16 [109], VGG19, Inception-V3 [100], Xception [110], ResNet50 [103], DenseNet121 [111], and CheXNet121 [112]. Wang et al., used a larger CWP dataset to investigate the potentiality of the deep learning model. They proposed only one CNN model, Inception-V3, for automated feature extraction and classification of pneumoconiosis in digital CXR, and compared this with the performances of two certified radiologists [70]. Recently, Devnath et al. [68] proposed an innovative method to detect CWP in CXR for a small dataset. They used a CNN model to extract multi-level and multidimensional features from the proposed architecture [112].

### 4.4. Detection Approach of CWP

This section discusses the feature classification methods used in the above literature. This study found three patterns of detection proposed in the CAD system of CWP. These are: classical methods, including computer and ILO-based detection, traditional machine learning-based detection, and deep learning (CNN-based) detection. The details of these approaches are outlined in the following sub-sections.

#### 4.4.1. Classical Methods

**Figure 4:**
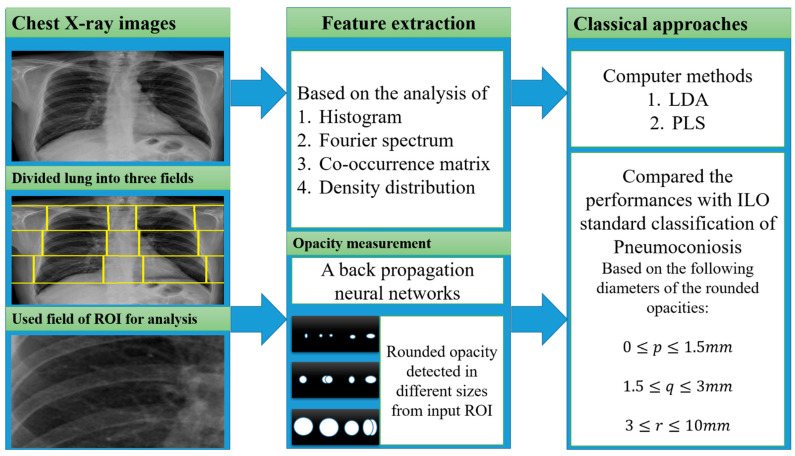
The illustration of the classical approaches was used for CWP detection.

In the past year, the texture features were mostly classified using classical computer-based methods and ILO-based standard classification [34,39,40,41,42,43,44,45,46,47,48], as shown in Figure 4. A linear discriminant analysis (LDA) and partial least squares (PLS) regression function has performed this in computer-based classification methods [34,44,45,46,47,48]. LDA and PLS are the classic statistical approaches for reducing the dimensions of characteristics to improve the classification. Besides this classification method, some researchers used the classical ILO standard-based guideline as shown in Figure 4. The profusion of small round opacities and ILO extent properties indicated normal and abnormal classes. Neural networks have been applied to find the shape and size of round opacities from ROI images [36,37,38,94]. The X-ray abnormalities were categorised and compared with the results of the standard ILO measurement of the size and shape of the round opacities, as in Figure 1.

A summary of all classic approaches corresponding to feature extractions with various inputs is shown in Figure 4. The performances of the classical methods are demonstrated in Table 2. The CAD performances of the 15 articles reviewed in Table 2 include classical methods with their corresponding data information and feature extraction methods. In the performance analysis of the classical methods, we found that the computer-based statistical approaches LDA and PLS were achieved overall accuracy with characteristics based on Fourier spectrum, histogram, and co-occurrence matrix analysis [44,47,48]. The descriptions of features are mentioned in Table 1.

#### 4.4.2. Traditional Machine Learning

**Figure 5:**
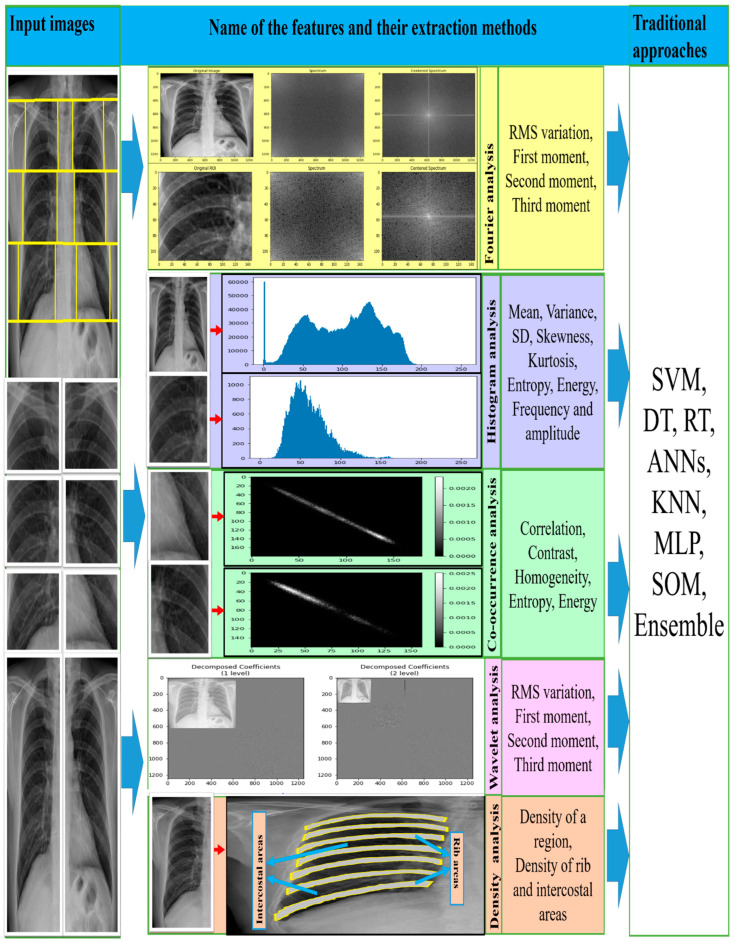
The illustration of the traditional approaches used for CWP detection.

Most texture features, from Fourier spectrum, co-occurrence matrix, histogram, wavelet transform, and density distribution, are classified using different traditional machine learning classifiers, namely support vector machines (SVM) [51,52,54,56,57,59,60,61,62,63], decision trees (DT) [52,56], random trees (RT) [51,54,60,62], artificial neural networks (ANNs) [53,55,58], K-nearest neighbors (KNN) [65], self-organizing maps (SOM) [65], backpropagation (BP), radial basis function (RBF) neural networks (NN) [51,54,60,62,65,66], and ensemble classifiers [52,59,61]. Figure 5 shows how the researchers connected various texture features with traditional machine learning classifiers to detect CWP in CXR. A set of features was derived from the corresponding transformation of various X-ray inputs. Transformation methods were discussed separately in the feature analysis section above. The abstracts of these features for CWP detection are described in Table 1.

This review found that SVM performed best compared to the other classifiers on ROI-based texture features, which also indicated that SVM with a radial basis function (RBF) kernel is more noticeable than linear and polynomial kernel functions. The maximum AUC (area under the curve) value of the receiver operating characteristic (ROC) curve indicated the SVM classifier’s ability to classify texture features. It was also seen that an ensemble of multiple classifiers would improve detection performance. In [52,59,61], the authors proposed an ensemble of multi-classifier and multi ROI decisions for the diagnosis of CWP, which improved the overall classification result. Details of the performance of individual machine learning classifiers and their overall learning have been demonstrated in Table 3.

The four feature extraction methods (Fourier spectrum, wavelet, histogram, and co-occurrence matrix analysis) outperformed classical approaches with the traditional machine learning classifiers. Table 3 summarises the CWP detection assessment performance based on the 18 reviewed articles that used different machine learning classifiers. Among all classifiers, the SVM exceeded the others in terms of histograms and co-occurrence characteristics of chest X-ray radiographs [59,61,63,64]. Moreover, SVM was used in the maximum and a bigger number of CWP data sets in the literature, demonstrating average accuracy, specificity, recall, and area under the curve (AUC).

#### 4.4.3. CNN-Based

**Figure 6:**
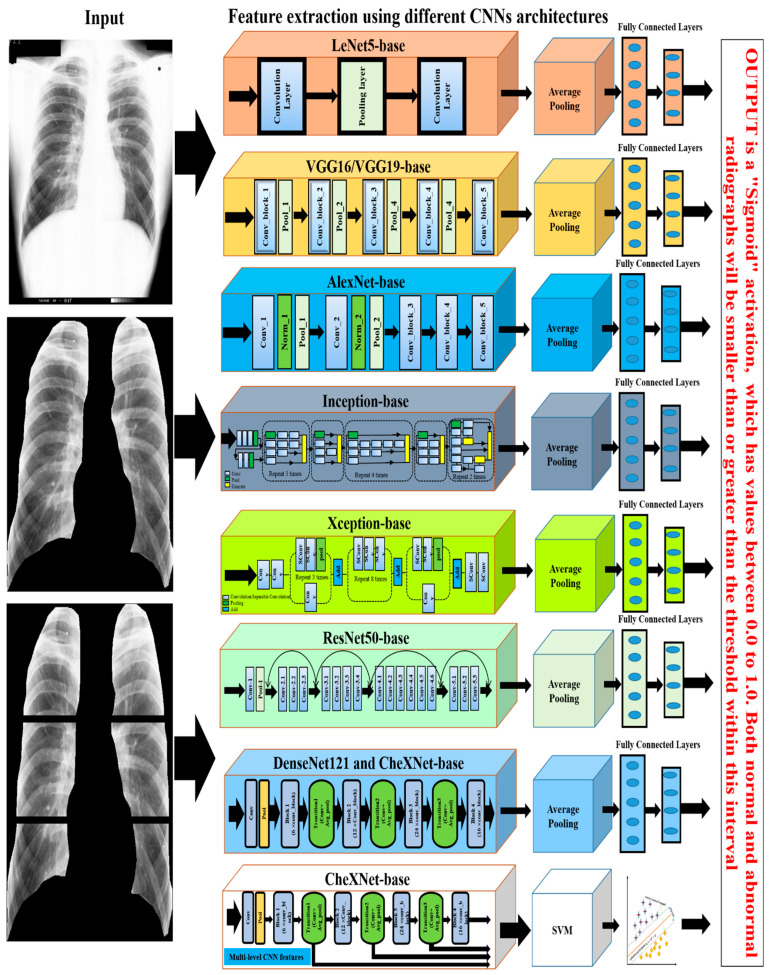
The illustration of the deep learning approaches used for CWP detection.

For the period 2019–2021, this review only found eights studies that proposed using deep convolutional neural network (CNN) models to classify CWP (black lung disease) in CXR [49,67,68,69,70,71,72,73]. They used different pre-trained deep learning models for non-textured feature extraction then applied a fully connected layer with binary classifier for normal or abnormal (black lung) classification. Over the past few years, various CNN models, such as VGG16 [109], VGG19, AlexNet [102], Inception [100], Xception [110], ResNet50 [103], DenseNet121 [111], and CheXNet121 [112], have been developed based on the ImageNet database classification results. Each CNN model consists of two main parts: the base (top-removed), and the other is called the top. The base part of the CNN model is used as an automatic deep feature extractor and consists of a set of convolutional, normalisation, and pooling layers. The top part is used as a deep classifier and consists of a number of dense layers that are fully connected to the outputs of the base part of the model, as shown in Figure 6.

Devnath et al., investigated the CNN classifier performance with and without deep transfer learning, which suggested that the transfer learning with the deep CNN technique will improve the classification of black lung disease with a small dataset [71,72]. Arzhaeva et al., show that CNN performed better than the statistical analysis methods, including texture features from ROIs and ILO standard classification of pneumoconiosis in CXR [49].

Zheng et al. [73] applied transfer learning of five CNN models, LeNet [101], AlexNet [102], and three versions of GoogleNet [100], for CAD of CWP in a CXR films dataset. They showed that the integrated GoogleNetCF performed better than others on their dataset. Zhang et al. [67] implemented the ResNet [103] model to categorise normal and different stages of pneumoconiosis using six subregions of the lung, as shown by an example in the left column of Figure 6. They verified the best CNN performance with two groups of expert radiologists. Wang et al. also verified the performance of the Inception-V3 model with two certified radiologists [70] and found that CNN is more efficient than human performance. More recently, Devnath et al. [68] proposed a novel method for CWP detection using multi-level features analysis from the CNN architecture as shown in the bottom section of Figure 6. They applied transfer learning of the CheXNet [112] model to extract miltidimensional deep features from the different levels of their architecture. They then used these features to the traditional machine learning classifier, SVM. This intregrated framework outperformed the state-of-art different traditional machine and deep learning methods.

Deep learning-based CAD approaches to CWP disease were demonstrated in Table 4, in which the performances from 8 reviewed articles are summarised. Non-texture features were extracted using different CNN approaches. Among all detection approaches, deep transfer learning of GoogleNet, ResNet, and CheXNet achieved an average accuracy of more than 92% in the detection of CWP from chest X-ray radiographs. Overall analysis revealed that deep learning methods outperformed other traditional and classical approaches in CWP detection.

## 5. Study Limitations and Future Directions for Research

The literature search was based on the search string indicated in Section 2.1. The authors acknowledge that there could be other keywords or phrases that might have been missed in our search string. The search was also limited to the 11 selected databases, based on other literature reviews conducted in domains related to computer science, information technology, engineering, medicine, clinical health study, and computer-aid diagnosis of medical imaging. This review’s findings suggest several important knowledge gaps and future directions in research on the diagnosis of CWP (black lung disease) in CXR.

### 5.1. Direction 1: Combination of All Private Datasets

This review noticed that the datasets used in the literature are not publicly available, except for the Japanese Society of Radiological Technology (JSRT) database. This indicates a need for a common dataset on which future researchers could benchmark and compare their system’s performance. A suggestion from the review would be to collect all datasets into a common public source to enable a large and common dataset resource for the future research of CWP.

### 5.2. Direction 2: Apply Deep Transfer Learning

Over the past few years, transfer learning has been widely used with deep learning applications that overcome data shortages and long-term training issues, and lead to lower generalisation errors. This review concluded that transfer learning of deep CNN would be the best approach to diagnosing of black lung in CXR. A suggestion is to apply transfer learning with different CNN models, to compare performance to select the best one.

### 5.3. Direction 3: Apply SVM on the Deep CNN Feature

The authors suggest applying a machine learning algorithm, especially the SVM, for feature classification based on past research. Although the feature classification could be either textured or non-textured, the non-textured feature classification is recommended. CNN has a special characteristic to producing a discriminative level of feature after every convolutional layer. The multi-level CNN features could be a good starting place to implement SVM to detection CWP.

### 5.4. Direction 4: Apply Ensemble Learning

This review found that the ensemble of multi-model decisions for multi-level detection has positively impacted CWP detection. There are different ensemble learning methods, such as prediction label voting, prediction probability voting, weights averaging, weights multiplying, and multi-model predictions voting. The authors suggest that to improve performance, ensemble learning should be tried.

## 6. Conclusions

This paper reviewed the literature on CWP detection published in the last 5 decades. To date, about 40 studies investigated CAD methods for detecting of pneumoconiosis. We classified and summarised the feature extraction and detection approaches utilized for CAD in CWP using chest X-ray radiographs. Most of these studies employed classical and traditional machine learning approaches. At the time of writing, eight studies employed deep learning approaches which outperformed other detection methods. The accessibility of a large pneumoconiosis database will be the ultimate key to developing of an automated screening system. This review discussed future research in CWP detection, especially the CNN-based method for improving CAD systems for the detection of different pneumoconiosis. This study also described five major categories of texture feature analysis methods, which are widely used in various machine learning applications.

## Figures and Tables

**Table 1:** Details of different texture features and their descriptions.

Feature Types	Features Name	Descriptions
Fourier spectrum-based	RMS variation	A measurement of the magnitude of lung texture
	First moment	Central tendency of lung texture
	Second moment	A measure of dispersion from the overall central tendency
	Third moment	A measure of the nature (coarse or fine) of the lung texture
Co-occurrence matrix-based	Correlation	Measurement of the relationship from different angles or directions between each pair of pixels on the image. Most of them used directions such as 0°, 45°, 90° and 135°
	Contrast or inertia	Contrast measurements of pixel intensity (greyscale tone or colour tone) using a pixel and its neighbor across the whole image
	Homogeneity	Measures the proximity of the pairs of pixels across the diagonal of the co-occurrence matrix. It should be elevated if the greyscale levels of all diagonal entries is similar
	Entropy	Measures spatial disturbances in pixel intensity relations which could be responsible for the image abnormality
	Energy	Shows the uniformity of the intensity relationships of the pixels by measuring the number of repeated pairs. The higher value of energy means the bigger homogeneity presents in the texture
Histogram-based	Mean	A measure of the colour intensity of each pixel on which the image brightness depends
	Variance	A measure of the breadth of the histogram indicates the deviation of the grey levels from the mean value
	SD	A scalar value computed from the image array that shows the lower or higher contrast of the colour intensities
	Skewness	The positive and negative asymmetry represents the degree of distortion of the histogram in relation to the mean intensity distribution, giving an idea about the image of a surface
	kurtosis	It is a measure of the degree of sharpness of the histogram relative to the mean intensity distribution
	Entropy	Entropy measures the random nature of the distribution of coefficient values on intensity distributions. It provides high readings with an image of more intensity levels
	Energy	The energy characteristic measures the uniform distribution of the intensity levels. It provides high readings with an image of fewer intensity levels
Wavelet transform-based	Energy	A wavelet coefficient is calculated from the distribution of grey level intensity in the sub-band images on a successive scale. The different energy levels of the sub-bands provide the differences in texture patterns
Density distribution-based	Density of a region	Measures how many pixels are contained in a particular region. The rapidly changing density of a region indicates the profusion of opacities
	Density of rib areas	Measures the mean of the pixel densities obtained from all the rib areas. The higher contrast occurs when the opacities appear around the edges of the ribs.
	Density of intercostal areas	Measures the average pixel densities for all intercostal areas. A higher contrast occurs when the opacities appear around the edges between the intercostal and rib areas

**Table 2:** Summary of classical approaches included studies.

Year and Country of Data	Ref No.	Feature Analysis Method	Classical Approaches	Number of CWP CXR	Evaluation Performance
					Accuracy
2009 (M)	[34]	Histogram analysis	Computer and ILO standard	11	AUC > 80.00%
2002	[35]	Opacity measurement	NN and ILO standard-based	1	-
2001	[36]	Opacity measurement	NN and ILO standard-based	1	
2001	[37]	Opacity measurement	NN and ILO standard-based	1	-
2000	[38]	Opacity measurement	NN and ILO standard-based	1	-
1997 (U)	[39]	Fourier spectrum	Computer and ILO standard-based	68	-
1990 (J)	[40]	Fourier spectrum	Computer and ILO standard-based	-	
1988 (J)	[41]	Opacity measurement	Computer and ILO standard-based	9	81.0%
1987 (J)	[42]	Co-occurrence matrix, density distribution	Computer and ILO standard-based	11	81.8%
1980 (U)	[43]	Opacity measurement	Computer and ILO standard-based	3	67%
1976 (U)	[44]	Fourier spectrum	Computer and ILO standard-based	141	82.9%
1976 (U)	[45]	Density Distribution	Computer and ILO standard-based	36	80.5%
1975 (U)	[46]	Density Distribution	Computer and ILO standard-based	36	80.5%
1975 (U)	[47]	Histogram analysis	Computer and ILO standard-based	38	84.0%
1974	[48]	Fourier spectrum, co-occurrence matrix	Computer and ILO standard-based	141	88.0%

**Table 3:** Summary of traditional machine learning approaches included studies.

Year and Country of D	Ref No.	Feature Analysis Method	Traditional Machine Learning Approaches	Number of CWP CXR	Evaluation Performance			
					Accuracy	Specificity	Recall	AUC
2019 (A)	[49]	Histogram analysis	SVM, MLP, NN	71	SVM = 73.17%	92.31%	73.30%	
					MLP = 71.11%	72.00%	70.00%	
					NN = 83.00%	85.00%	82.00%	
2017 (J)	[50]	Fourier spectrum, co-occurrence matrix, histogram analysis	ANN	46	-	Category 1 = 38.2%	-	Category 1 = 73.0%
						Category 2 = 52.5%		Category 2 = 79.0%
						Category 3 = 60.1%		Category 3 = 85.0%
2014 (J)	[51]	Density distribution	SVM, RT, NN	15 right-lung	-	-	RT = 93.2%	-
							NN = 93.2%	
							SVM = 93.2%	
2014 (C)	[52]	Wavelet analysis	SVM and ensemble	40	90.5%	93.3%	84.9%	96.1%
2014 (J)	[53]	Fourier spectrum, co-occurrence matrix	ANN	15	-	-	-	93.0%
2013 (J)	[54]	Density Distribution	SVM, RT, NN	12 right-lung	-	-	RT = 91.67%	
							NN = 91.67%	
							SVM = 100.0%	
2013 (C)	[55]	Co-occurrence matrix, histogram analysis	ANN	40	79.3%	70.6%	91.7%	85.8%
2013 (C)	[56]	Wavelet analysis	SVM and DT	40	SVM = 87.2%	SVM = 90.6%	SVM = 80.0%	SVM = 94.0%
					DT = 83.2%	DT = 89.4%	DT = 70.0%	DT = 86.0%
2011 (J)	[57]	Co-occurrence matrix	SVM	68	69.7%	-	-	-
2011 (J)	[58]	Fourier spectrum, co-occurrence matrix	ANN	12	-	-	-	97.2%
2011 (C)	[59]	Co-occurrence matrix, histogram analysis	SVM and ensemble	250	88.9%	87.7%	92.0%	97.8%
2010 (J)	[60]	Density distribution	SVM, RT, NN	6 right-lung	-	-	-	-
2010 (C)	[61]	Co-occurrence matrix, histogram analysis	SVM and Classifiers ensemble	259	92.83%	90.25%	96.65%	-
2009 (J)	[62]	Density distribution	SVM, RT, NN	6 right-lung	-	-	-	-
2009 (C)	[63]	Histogram analysis	SVM	196	94.1%	94.6%	93.6%	
2009 (C)	[64]	Co-occurrence matrix	SVM	59	95.15%	94.2%	95.6%	
2002 (M)	[65]	Co-occurrence and spatial dependence matrix analysis	SOM, NN, KNN	74	SOM = 71.0%	-	-	
					NN = 75.0%			
					KNN = 72.0%			
2001 (C)	[66]	Co-occurrence matrix	NN	212	86.8%	-	-	-

**Table 4:** Summary of deep learning approaches included studies.

Year and Country of Data	Ref No.	Feature Analysis Method	Deep Learning Approaches	Number of CWP CXR	Evaluation Performance			
					Accuracy	Specificity	Recall	AUC
2021(C)	[67]	Non-texture CNN	ResNet	512	92.70%	-	-	-
2021(A)	[68]	Non-texture CNN	CheXNet	71	92.68%	83.33%	100%	97.05%
2020(A)	[69]	Non-texture CNN	Cascaded Learning, CheXNet	71	Cascaded = 90.24%	88.46%	93.33%	-
					CheXNet = 78.05%	80.77%	73.33%	
2020 (C)	[70]	Non-texture CNN	InceptionV3	923	-	93.30%	62.30%	87.80%
2020 (A)	[71]	Non-texture CNN	VGG16, VGG19, ResNet, InceptionV3, Xception, DenseNet, CheXNet	71	VGG16 = 82.93%	80.00%	84.62%	-
					VGG19 = 80.49%	80.00%	80.77%	
					ResNet = 85.37%	80.00%	88.46%	
					InceptionV3 = 87.80%	86.67%	88.46%	
					Xception = 85.37%	93.33%	80.77%	
					DenseNet = 82.93%	80.00%	84.62%	
					CheXNet = 85.37%	93.33%	80.77%	
2019 (A)	[49]	Non-texture CNN	15 layers CNN	71	90.24%	89.29%	90.74%	-
2019 (A)	[72]	Non-texture CNN	DenseNet, CheXNet	71	CheXNet = 85.37%	80.00%	88.46%	-
					DenseNet = 80.49%	73.33%	84.62%	
2019 (C)	[73]	Non-texture CNN	LeNet, AleXNet, InceptionV1, InceptionV2, GoogleNetCF	1600	GoogleNetCF = 93.88%	-	-	-
					InceptionV1 = 91.60%			
					InceptionV2 = 90.70%			
					AleXNet = 87.90%			
					LeNet = 71.6%			

## Data Availability

Not applicable.
